# Supplementary material for: Pan-genome analysis of six Paracoccus type strain genomes reveal lifestyle traits
Source: PLoS One. 2023 Dec 20;18(12):e0287947. doi: 10.1371/journal.pone.0287947 (PMC10732464; doi:10.1371/journal.pone.0287947)
Supplement: S1 Fig — The trees were reconstructed based on (A) 16S rRNA comparison using TYGS, and (B) WGS comparison with all 64 available Paracoccus genomes using ANI. Presented Paracoccus genomes used in this study are highlighted in red. Complete genomes are highlighted in blue. (PDF) [file pone.0287947.s001.pdf]

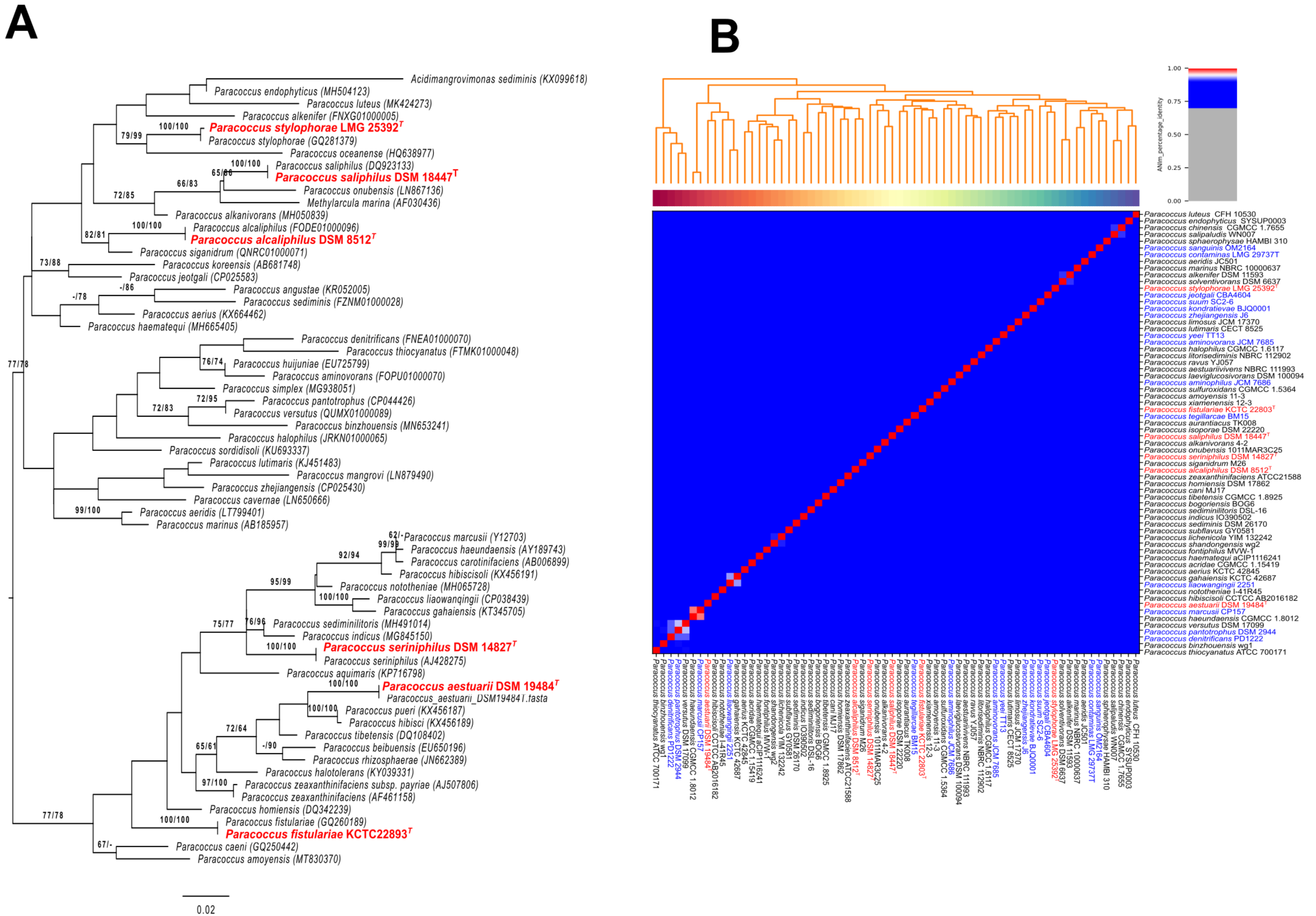

S1 Fig. Phylogenetic tree of investigated six *Paracoccus* type strains. The trees were reconstructed based on (A) 16S rRNA comparison using TYGS, and (B) WGS comparison with all 64 available *Paracoccus* genomes using ANI. Presented *Paracoccus* genomes used in this study are highlighted in red. Complete genomes are highlighted in blue.
